# Social media use and health promotion among cancer survivors

**DOI:** 10.1002/pon.6299

**Published:** 2024-01

**Authors:** Joseph O. Atarere, Henry K. Onyeaka, Onyema G. Chido-Amajuoyi, Comfort Adewunmi, Chisom Nwaneki, Gideon T. Dosunmu, Chilotam O. Faith, Hermioni L. Amonoo

**Affiliations:** 1 Department of Medicine, MedStar Union Memorial Hospital, Baltimore, Maryland, USA; 2 Harvard Medical School, Boston, Massachusetts, USA; 3 Department of Psychiatry, Massachusetts General Hospital, Boston, Massachusetts, USA; 4 Department of Epidemiology, The University of Texas MD Anderson Cancer Center, Houston, Texas, USA; 5 Division of Geriatrics and Gerontology, Emory University School of Medicine, Atlanta, Georgia, USA; 6 Department of Medicine, Saint Peter’s University Hospital, New Brunswick, New Jersey, USA; 7 Department of Hematology and Oncology, University of Chicago, Chicago, Illinois, USA; 8 Department of Medical and Laboratory Sciences, Madonna University, Madonna, Nigeria; 9 Department of Psychosocial Oncology, Dana-Farber Cancer Institute, Boston, Massachusetts, USA; 10 Department of Psychiatry, Brigham and Women’s Hospital, Boston, Massachusetts, USA

**Keywords:** cancer, cancer survivors, health behaviors, health promotion, oncology, social media, sociodemographic

## Abstract

**Objective:**

Social media is becoming recognized as an effective platform for cancer health promotion, education, care, and support. However, its utility as a health promotion tool remains relatively unexplored.

**Methods:**

Using cross-sectional data from the 2017–2020 Health Information National Trends Survey, we evaluated health-related usage of social media among cancer survivors and individuals without a history of cancer. We also examined the participant characteristics associated with social media usage and evaluated the relationship between social media use and positive health behaviors among the cancer survivors.

**Results:**

Overall, cancer survivors (*n* = 2579) were as likely as individuals without a history of cancer to use social media for health promotion. Males [OR 0.65; 95% CI (0.45, 0.93)] and older adults (>60 years old) [OR 0.27; 95% CI(0.10, 0.77)] were less likely to use social media, while higher income [OR 2.27; 95% CI (1.05, 4.92) middle income; OR 1.90; 95% CI (1.17, 3.09) high income] and educational levels [OR 3.29; 95% CI (1.85, 5.84) some college; OR 2.36; 95% CI (1.30, 4.28) college graduate or more] were associated with more health-related social media use among survivors. Cancer survivors used social media for online support groups more than other individuals, and those who used at least one form of social media for health-related purposes increasingly meet national recommendations for strength training compared to non-users [OR 2.15; 95% CI (1.48, 3.13)].

**Conclusions:**

Our findings demonstrate the potential utility of social media to promote positive health behaviors among cancer survivors. Further research is needed to describe the efficacy of social media-based interventions for improving health behaviors in diverse cancer populations.

## 1 | BACKGROUND

The number of cancer survivors in the United States (US) continues to increase with 18 million reported in 2022^1^ and this number is projected to reach 22 million by 2030.^1^ While secondary cancer prevention contributes the most to the growing number of survivors, improved diagnostic tools, public health surveillance measures, and advancements in cancer therapeutics are also implicated.^1^ However, many cancer survivors experience a diverse range of unmet physical and psychological needs such as, sleep difficulties, chronic pain, fatigue, psychological distress, and treatment-related adverse effects which negatively impact overall quality of life (QOL).^2^ Further, chronic diseases including cardiovascular diseases cause greater morbidity and mortality in cancer survivors compared to individuals without a history of cancer.^3,4^

Lifestyle interventions effectively promote physical and psychosocial wellbeing in diverse clinical populations.^4^ Indeed, modifiable positive lifestyle behaviors (e.g., physical activity and diet) improve survival in diverse patients with cancer.^5^ For example, physical activity and high-quality diets are associated with better psychosocial outcomes, QOL, physical function, and overall survival in cancer survivors.^5,6^ Accordingly, the American Cancer Society (ACS) has recommended the robust inclusion of health behaviors (e.g., physical activity, nutrition, and diet) in routine cancer care.^6^ However, the adoption of healthy lifestyle behaviors in cancer care remains suboptimal and is generally lower than nationally recommended guidelines.^7^ Hence, newer, innovative, and readily accessible interventions that promote and support positive health behaviors in cancer survivors are crucial.

With the ubiquity and increased affordability of mobile devices and broadband Internet networks, about half of the global population use social media (SM) platforms.^8^ As such, SM platforms like YouTube, Tik Tok, Meta (formerly FaceBook), and X (formerly Twitter) have emerged as effective tools for communication, making social connections, sharing information, and exchanging ideas across diverse fields.^8,9^ The use of SM for health education and promotion has received attention due to its ability to remove physical barriers limiting access of health interventions to disadvantaged populations (e.g. elderly, low income, rural dwellers, etc.)^10–12^ but evidence of its benefit in health promotion has, however, been mixed.^13,14^ For example, while a 2017 systematic review found no effect of SM interventions on smoking cessation, a 2019 meta-analysis found a positive effect of SM interventions on smoking cessation.^13,14^

In preventive health, SM has been shown to help improve screening and early detection of breast, lung, and colorectal cancer.^15^ Studies have also shown several benefits to the use of SM among cancer survivors. For example, in a recent systematic review, Gentile et. al succinctly outlined five broad benefits of SM throughout the cancer journey: facilitating patient engagement and empowerment, psychosocial support, enhanced patient–physician relationship, informational support, and cancer-related research education.^16^ The use of SM helps overcome barriers of in-person cancer support groups like occupational and family responsibilities, and transportation difficulties.^17^ The support provided by these platforms has been associated with reduced social isolation and improved health self-efficacy.^18^

SM has been associated with increased QOL, decreased psychological burden, and increased knowledge of preventive health among cancer survivors.^19^ In another systematic review, Ghahramani et al explored the benefits of social media beyond creating awareness, highlighting its usefulness as a tool in increasing engagement in health promotion programs.^20^ Thus, SM has promise and affords new opportunities to support lifestyle and behavioral modifications for cancer survivors. In fact, SM has been used to support the implementation of programs that improve the physical health of patients with medical conditions like depression and anxiety.^21^ However, it remains underutilized for the implementation of behavioral health interventions among cancer survivors.

Data on the availability, usage, potential impact, and sociodemographic factors associated with usage of SM tools for health purposes at the population level are limited for cancer survivors. A nuanced understanding of the facilitators of cancer survivors’ usage of SM tools to manage their health is critical to the implementation of large-scale SM interventions which promote healthy behaviors in cancer patients and survivors. Hence, we used data from the 2017 to 2020 Health Information National Trends Survey to: (i) examine the usage of SM among cancer survivors compared to individuals without a history of cancer, (ii) examine the trends in SM utilization among cancer survivors, and (iii) evaluate the association between SM usage and the adoption of healthy lifestyle behaviors in cancer survivors.

## 2 | METHODS

### 2.1 | Data source

We pooled data from the fifth iteration of the National Cancer Institute’s Health Information National Trends Survey (HINTS 5). HINTS is a survey that nationally represents the US adult population (18+ years). It has been conducted every 1–3 years, since 2003, and was created to assess the public’s understanding and use of cancer information by documenting cancer history, health behaviors, and communication technology, including the use of SM.

We collected data from January to May 2017 (HINTS-5, cycle-1), January to May 2018 (HINTS-5, cycle-2), January to May 2019 (HINTS-5, cycle-3) and February to June 2020 (HINTS-5, cycle-4). The response rates were 32.4% for cycle-1, 32.9% for cycle-2, 30.3% for cycle-3, and 36.7% for cycle-4. Complete information about data collection and methodologies including the sampling and weighting process of the HINTS datasets is described in their corresponding methodology reports.^22^ Briefly, HINTS-5 employed a two-stage, stratified random sampling technique where non-vacant residential addresses obtained from the Marketing Systems Group (MSG) were examined for an adult from each household who was selected to participate in the survey using the ‘Next Birthday’ method. This stratification was done to increase precision of minority subpopulation estimates.

### 2.2 | Study population

All adults (≥18 years) were included in this study. Cancer survivors were determined by evaluating responses (Yes or No) to the question, “Have you ever been diagnosed as having cancer?” Those who answered “Yes” were classified as cancer survivors.^23^ All study participants provided written informed consent. The data is de-identified and classified as exempt from review by the US National Institutes of Health, Office of Human Subjects Research Protections. We followed the Strengthening the Reporting of Observational Studies in Epidemiology (STROBE) reporting guidelines.^22,24^

### 2.3 | Measures

#### 2.3.1 | Social media use

The primary outcome in this study was SM use among cancer survivors within the past 12 months preceding the survey. We examined responses (Yes or No) to the following survey questions:

General SM use was ascertained by participant responses to the question: “In the last 12 months, have you visited a social networking site such as Meta or LinkedIn?”

Health-related SM usage was derived in response to the following survey questions: “In the last 12 months, (i) have you shared health information on social networking sites, such as Meta or X? (ii) have you participated in an online forum or support group for people with similar health or medical issues? and (iii) have you watched a health-related video on YouTube?”

Additionally, we created a composite binary variable to characterize health-related SM use. Participants who endorsed at least one or more health-related SM use were categorized as SM users and those reporting no health-related SM use were characterized as non-users.

#### 2.3.2 | Social media use for health behavior change in cancer survivors

Participants’ reports of achieving the nationally recommended levels of (i) general physical activity (150+ minutes per week), (ii) sedentary activity (<8 h), (iii) 2+ days of strength and exercise training and (iv) fruit and vegetable intake (2+ cups of each, per day) were assessed.

#### 2.3.3 | Covariates

We included self-reported age, race, sex, educational level, household income, health insurance coverage, area of residence, comorbidities, personal history of cancer, and access to a primary care provider as done in previous studies.^25,26^

#### 2.3.4 | Statistical analysis

All analyses were conducted using Stata 17.0 statistical software (StataCorp LP, College Station, Texas, USA). All tests were two-sided and p values < 0.05 were considered statistically significant. Final person weights and jack-knife replicate weights provided within the HINTS-5 dataset were used to provide national estimates representative of the US population. We described the demographic characteristics of the study population by cancer survivor status using chi-squared tests, presenting values in weighted percentages. We also described the characteristics of the cancer survivors by SM use with chi-squared tests. Using separate logistic regression models adjusted for sociodemographic factors, we compared the odds of SM use between cancer survivors and those without a history of cancer.

In investigating the relationship between health-related SM use and positive lifestyle behaviors, we only included cancer survivors who demonstrated curiosity in looking for cancer information as there are a variety of health-related uses of SM. Curiosity in seeking out cancer information was determined by evaluating the responses (Yes or No) to the question, “Have you ever looked for information about cancer from any source?” Only those who responded “yes” to this question were included in the analysis. Using separate multi[sp]-variable logistic regression models adjusted for sociodemographic characteristics, we examined the relationship between SM use and positive lifestyle behaviors (adhering to recommendations for physical activity and diet) among cancer survivors.

## 3 | RESULTS

### 3.1 | Population characteristics

Of the 15,871 participants included in the study, 2579 (16.2%) were cancer survivors. A greater proportion of cancer survivors were female (57.1% vs. 50.3%; *p* = 0.001) and non-Hispanic whites (79.3% vs. 62.9%; *p* < 0.0001) compared to those without a history of cancer. 62.3% of cancer survivors were at least 60 years of age and over 90% were 40 years of age or older. Among cancer survivors, the most reported cancer diagnoses were skin cancer (40.9%), breast cancer (17.6%), prostate cancer (10.0%), cervical cancer (8.6%), and colorectal cancer (6.5%). Further details on the sociodemographic characteristics of the study population are shown in Table 1.

### 3.2 | Use of social media among cancer survivors

Among cancer survivors, there was a gradual increase in the proportion of respondents who reported visiting a social networking site from 2017 to 2020 but not for sharing health information, participating in online support groups, or watching health related videos on YouTube (Supplement Figure 1). About a third (36.1%) of cancer survivors reported at least one health-related use of SM in the last 12 months. Watching health-related videos on YouTube was the most frequent use of SM among cancer survivors (Table 2). The sociodemographic characteristics of cancer survivors by SM use are shown in Supplement Table 1.

After adjusting for sociodemographic differences, cancer survivors were just as likely as individuals without a history of cancer to have visited social networking sites [OR 0.95; 95% CI (0.79, 1.15)], shared health information on SM [OR 1.09; 95% CI (0.85, 1.40)], and watched YouTube videos [OR 1.01; 95% CI (0.83, 1.21)]. They were however more likely to participate in online support groups [OR 1.65; 95% CI (1.12, 2.41)] than individuals without a history of cancer (Table 2).

Among cancer survivors, males [OR 0.65; 95% CI (0.45, 0.93)] and older adults [OR 0.27; 95% CI (0.10, 0.77)] were less likely to use SM while higher income and educational levels were associated with greater levels of SM use. Importantly, Hispanics [OR 1.87; 95% CI (0.82, 4.28)] and Blacks [OR 1.94; 95% CI (0.72, 5.23)] were just as likely to use SM as non-Hispanic whites (Table 3).

### 3.3 | Association between social media use and health promotion among cancer survivors

Table 4 shows the results of multivariable logistic regression for the association between health-related SM use with positive lifestyle behaviors among cancer survivors. Cancer survivors who used at least one form of SM for health-related purposes in the past 12 months were more likely to meet national recommendations for strength training (>2 days/week) compared to non-users [OR 2.15; 95% CI (1.48, 3.13)]. There was, however, no association between health-related SM usage and meeting national recommendations for weekly physical activity [OR 1.13; 95% CI (0.78, 1.63)], daily sedentary time [OR 1.18; 95% CI (0.83, 1.68)] or fruit [OR 1.40; 95% CI (0.66, 2.97)], and vegetable [OR 1.16; 95% CI (0.63, 2.13)] intake among cancer survivors.

## 4 | DISCUSSION

This study is among the first to use a large, diverse sample of US adults to examine the use and potential impact of SM in health behavior promotion for cancer survivors. Specifically, we evaluated the prevalence and trends in health-related SM usage, the sociodemographic factors associated with health-related SM usage, and the association between SM usage in cancer survivors with their current health behaviors (e.g., physical activity and diet), drawing from a nationally representative sample of cancer survivors in the US.

Social media sites like Meta and X have increased in popularity over the past decade^27^ but there is limited data describing use of SM by US cancer survivors. Although the majority of cancer survivors (~60%) use SM, only a small minority use SM for health-related purposes, that is, 11.9% (sharing health information), 27.0% (watch health-related videos on YouTube), and 8.5% (participating in online support groups), respectively. These rates are however comparable or higher (in the case of online support group participation) than rates of health-related SM use among individuals without a history of cancer. Given the established association between SM use and improved QOL among cancer survivors,^19^ quantitative and qualitative studies are needed to provide more insights on the barriers and facilitators to SM use for health-related purposes among cancer survivors.

We show that cancer survivors were more likely to participate in online support groups than individuals without a cancer history. Historically, online support groups are considered a safe and comfortable environment for information seeking among cancer survivors.^28^ Cancer support groups have a high amount of user-generated contributions^16^ where disease and therapy-related information shared among cancer survivors may alleviate anxiety.^28^ This became particularly important during the COVID-19 pandemic as fear of infection and social restrictions caused heightened levels of anxiety among cancer survivors.^29^ Thus, our results highlight the potential impact of SM for social support among cancer survivors beyond traditional clinic settings which should be further studied in larger prospective studies.

Although SM use between cancer survivors and individuals without a cancer history are comparable, male sex, older age, and lower socioeconomic status were associated with less SM use for health-related purposes. These participant characteristics, such as male sex, are associated with higher cancer mortality likely mediated by poor health behaviors like current or previous cigarette smoking and excessive consumption of alcohol which are more common among male cancer survivors.^30,31^ Similarly, lower income and educational attainment were associated with less SM use for health-related purposes, consistent with previous studies among cancer survivors.^32^ Educational attainment positively correlates with digital literacy among cancer survivors.^33^ Low digital literacy negatively impacts health information interpretation on SM especially for patients with limited English proficiency, resulting in reduced trust and use of online health information.^34^ X recently included a fact-checking feature to help address misinformation on its platform.^35^ Such features that combat misinformation and more user friendly SM interfaces in English and other languages, may increase the use of SM for health-related needs among cancer survivors with low health literacy.

Certain cancers (e.g., lung cancer) are associated with social stigma and isolation due to negative perceptions of the causal link between behavior (e.g., cigarette smoking) and cancer risk.^36^ SM provides an opportunity for cancer survivors to join affinity groups which may motivate and foster support for engagement in positive lifestyle behaviors like smoking cessation. Our findings on the association between SM use and health behaviors were mixed. While SM use for health purposes was associated with increased adherence to national guidelines for strength training among cancer survivors, there was no association between SM use and national physical activity or dietary targets. Although strength training and physical activity are not synonymous, strength training has been associated with increased physical fitness among breast cancer survivors.^37^ A social media-based intervention on Meta found a positive relationship between SM engagement and physical activity levels among young adult cancer survivors.^38^ A meta-analysis however failed to find any effect of SM use on physical activity levels.^39^ Considering the complexities of implementing behavior change interventions, our findings suggest that health-related SM use may promote certain healthy behaviors like physical activity among adult cancer survivors. There are however several limitations with the use of SM for health promotion. First, the algorithms on many SM platforms are designed to show users the most engaging content which may not necessarily be useful to them.^40^ Second, most SM platforms are unregulated and many online sources provide incomplete or misleading health information intended to create dread or provide unfounded hope for cure of a terminal illness.^16,41^ The spread of misinformation is aided by SM echo chambers, the ease of transmitting information and the naivety of new SM users.^41,42^ There is also a lot of cancer information on SM which are anxiety provoking and can lead to prolonged discussions of unverified information during regular clinic visits. Additionally, there are a lot of opportunists on SM who target vulnerable cancer survivors with false claims of expensive curative cancer therapies.^16^ These barriers may limit the use of SM to induce positive behavior change among cancer survivors. Future efficacy trials should explore the impact of SM based supportive interventions on diverse health behaviors for cancer survivors.

### 4.1 | Study limitations

There are several limitations worth discussing. First, the cross-sectional nature of the data used in this study introduces the possibility of residual confounding preventing us from drawing causal conclusions from our findings. Similarly, there is a possibility that cancer survivors who engage in less strength training may be more socially isolated and less likely to use SM, hence reverse causality cannot be ruled out. Second, the outcomes in this study are based on self-reported data and the results of our analysis are subject to recall bias by the survey respondents. Third, the survey questions in the HINTS dataset limited our ability to ascertain the number of SM platforms used by cancer survivors, the specific reason for use, frequency, and duration of use. Our definitions of SM use have, however, been utilized in previous studies using national surveys.^43^ Fourth, we were unable to assess the current status of cancer diagnosis (current or previous), stage of cancer and whether participants were receiving treatment for their cancer or not. Finally, only individuals with home addresses could be included in this study. Our findings are thus not generalizable to homeless cancer survivors who may benefit from the social support provided by SM.

### 4.2 | Clinical implications

Although positive lifestyle behaviors like physical activity and maintaining a healthy diet are recommended as part of routine cancer care by the ACS, their uptake among cancer survivors is low. Since many cancer survivors use SM, our findings highlight the potential impact of SM as a vehicle to promote positive behavioral change among cancer survivors. However, for these tools to be implemented on a large scale, sociodemographic disparities in SM use should be addressed.

In summary, our study demonstrates the utility of SM for improving health among cancer survivors by fostering the development of groups for psychosocial support and enhancing positive health behaviors. Our study further highlights the need for prospective and randomized studies to describe the efficacy of SM tools for improving health behaviors in cancer survivors, especially those from lower socioeconomic backgrounds.

## Supplementary Material

Supplement 1

Additional supporting information can be found online in the Supporting Information section at the end of this article.

## Figures and Tables

**TABLE 1:** Bivariate association of sociodemographic characteristics by cancer status.

Sociodemographic characteristics	Total (*n* = 15,871)%^a^	No cancer history (*n* = 13,292)%^a^	Cancer survivor (*n* = 2579)%^a^	Chi-squared statistic	*p*-value
Gender					
Female	50.93	50.32	57.05	15.4311	0.0001
Male	49.07	49.68	42.95		

Age					
Young (<40 years)	32.78	35.19	8.96	219.0423	<0.0001
Middle aged (40–59 years)	39.86	40.98	28.73		
Older (60+ years)	27.36	23.83	62.31		

Race					
Non-Hispanic white	64.32	62.85	79.26	27.0181	<0.0001
Non-Hispanic black	10.86	11.10	8.40		
Hispanic	16.39	17.15	8.61		
Other^b^	8.43	8.90	3.73		

Education					
High school or less	30.92	30.60	34.07	3.4688	0.0339
Some college	38.00	38.02	37.84		
College graduate or postgraduate	31.08	31.38	28.09		

Income group					
Low (<$35,000)	28.69	28.43	31.42	3.5096	0.0331
Middle ($35,000 to < $50,000)	13.62	13.42	15.70		
High ($50,000+)	57.68	58.15	52.88		

Insurance status					
Uninsured	8.54	9.04	3.55	22.5581	<0.0001
Insured	91.46	90.96	96.45		

Comorbidities					
None	52.72	54.50	35.09	126.9251	<0.0001
At least one	47.28	45.50	64.91		

Residence					
Urban	86.75	87.00	84.26	5.9726	0.0154
Rural	13.25	13.00	15.74		

Access to a primary care provider					
No	35.47	37.42	16.00	205.1571	<0.0001
Yes	64.53	62.58	84.00		

a All numbers in the table are weighted column percentages.

b Other race includes non-Hispanic multiracial, non-Hispanic Asian, non-Hispanic American Indian or Alaskan native, Native Hawaiian or Pacific Islander.

**TABLE 2:** Odds of social media use by cancer status.

Social media variable	Total (*n* = 15,871)%^a^	No cancer history (*n* = 13,292)%^a^	Cancer survivor (*n* = 2579)%^a^	Adjusted OR^b^, [95% CI]	*p*-value
Visit social networking site	70.79	72.00	58.81	0.95 [0.79, 1.15]	0.603
Share health information on social network sites	14.45	14.71	11.86	1.09 [0.85, 1.40]	0.502
Participate in online support group	7.59	7.50	8.53	1.65 [1.12, 2.41]	0.011
Watch health-related youTube videos	35.90	36.79	27.03	1.01 [0.83, 1.21]	0.955

a All numbers in the table are weighted column percentages.

b Analysis was adjusted for age, gender, race, educational level, household income, having a regular provider, presence of comorbidities, and rural-urban residence.

**TABLE 3:** Factors associated with social media usage among cancer survivors.

Sociodemographic variables	Cancer survivors adjusted OR^a^, 95% CI	Chi-squared statistic	*p*-value
Male	0.65 [0.45, 0.93]	−2.34	0.020

Age group			
Middle aged (40–59 years)	0.74 [0.26, 2.06]	−0.59	0.559
Older (60+ years)	0.27 [0.10, 0.77]	−2.48	0.014

Racial group			
Non-Hispanic black	1.94 [0.72, 5.23]	1.31	0.191
Hispanic	1.87 [0.82, 4.28]	1.49	0.137
Other^b^	2.16 [1.02, 4.55]	2.03	0.043

Income level			
Middle ($35,000 to < $50,000)	2.27 [1.05, 4.92]	2.10	0.037
High ($50,000+)	1.90 [1.17, 3.09]	2.60	0.010

Educational level			
Some college	3.29 [1.85, 5.84]	4.09	<0.0001
College graduate or more	2.36 [1.30, 4.28]	2.85	0.005
Rural residence	0.75 [0.44, 1.28]	−1.05	0.297
Comorbidity	0.99 [0.65, 1.51]	−0.05	0.963
Insured	0.79 [0.27, 2.31]	−0.43	0.666
Primary care provider access	1.68 [0.91, 3.11]	1.66	0.098

*Note*: The outcome modeled is ‘social media use’.

a The model is adjusted for gender, age, race, income level, educational level, area of residence, comorbidity, insurance status and primary care provider access.

b Other race includes non-Hispanic multiracial, non-Hispanic Asian, non-Hispanic American Indian or Alaskan native, Native Hawaiian or Pacific Islander.

**TABLE 4:** Association between social media use and health promotion among cancer survivors.

Social media variable	Total (*n* = 1840)%^a^	Social media non-users (*n* = 1175)%^a^	Social media users (*n* = 665)%^a^	Social media adjusted OR^b^, [95% CI]	*p*-value
Strength training (>2 days/week)	36.97	31.93	44.46	2.15 [1.48, 3.13]	<0.0001
Physical activity levels (>150 min/week)	51.51	51.24	51.96	1.13 [0.78, 1.63]	0.520
Daily sedentary time (>8 h sitting daily)	58.05	55.71	61.87	1.18 [0.83, 1.68]	0.349
Fruit intake (2+ cups daily)^c^	19.11	17.32	22.03	1.40 [0.66, 2.97]	0.382
Vegetable intake (2+ cups daily)^c^	25.10	24.17	26.62	1.16 [0.63, 2.13]	0.642

a All numbers in the table are weighted column percentages.

b Analysis was adjusted for age, gender, race, educational level, household income, having a regular provider, presence of comorbidities, and rural-urban residence.

c Data available for only 2017 and 2019.

## Data Availability

The data that support the findings of this study are openly available in National Cancer Institute HINTS at https://hints.cancer.gov/.
