# Supplementary material for: Social media use and health promotion among cancer survivors
Source: Psychooncology. Author manuscript; Available in PMC 2026 Jul 30. (PMC13420014; doi:10.1002/pon.6299)
Supplement: Supplement 1 [file NIHMS2195893-supplement-Supplement_1.docx]

**Supplement Figure 1: 2017 to 2020 Trends in Health-Related Social Media Use by Cancer Survivor Status**


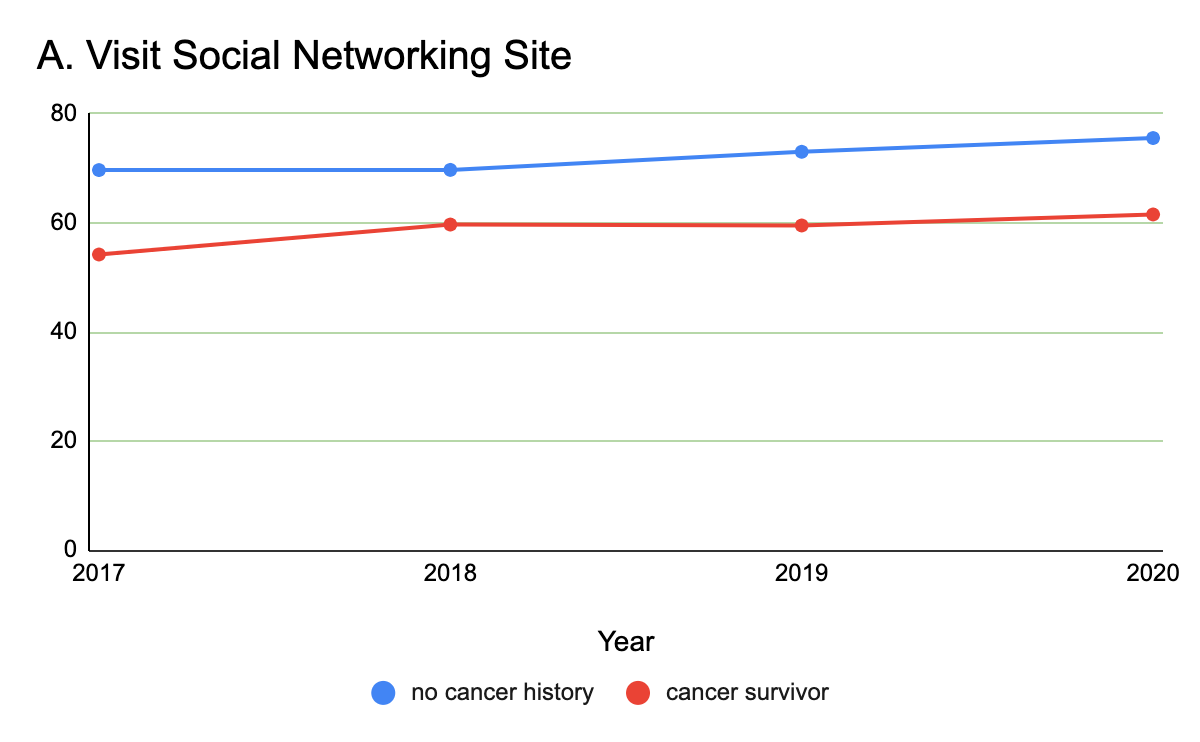

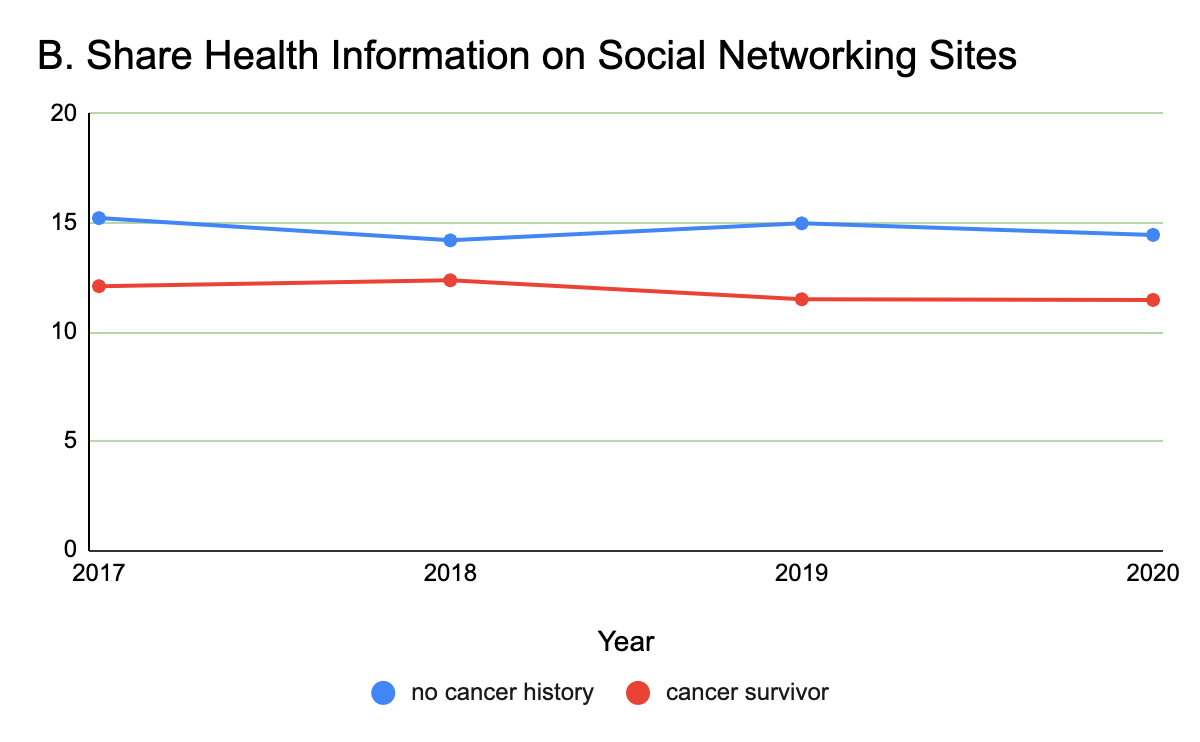


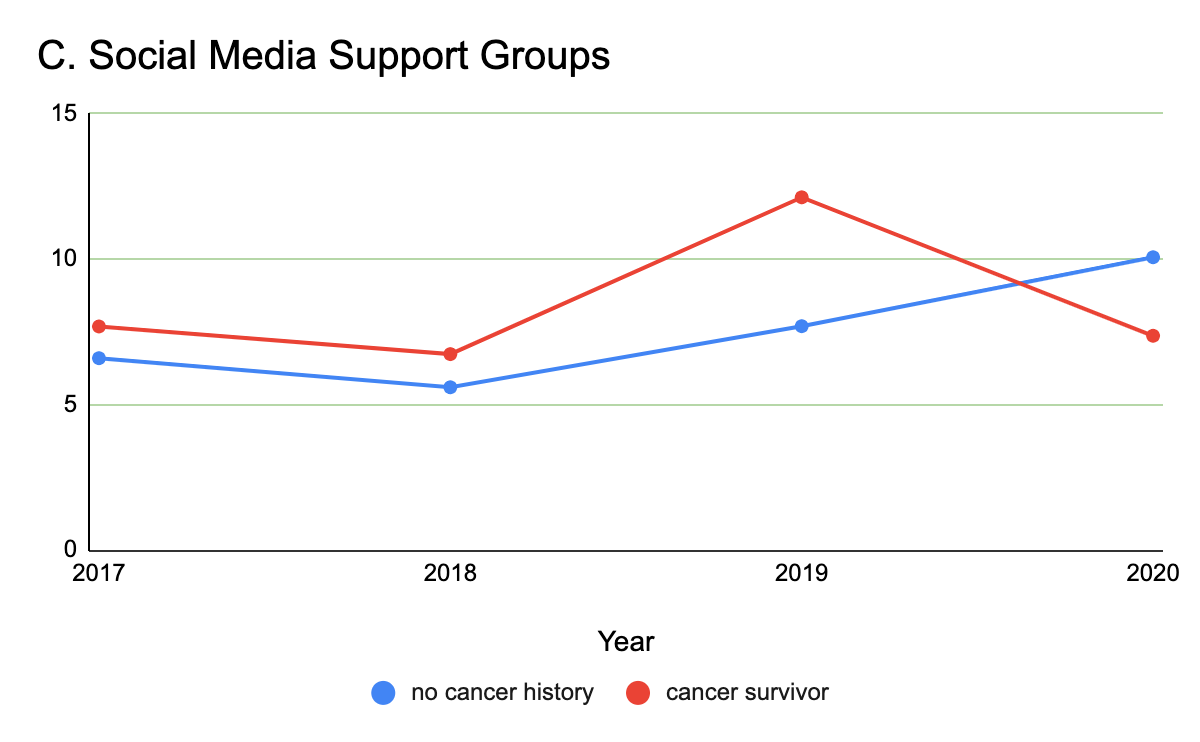

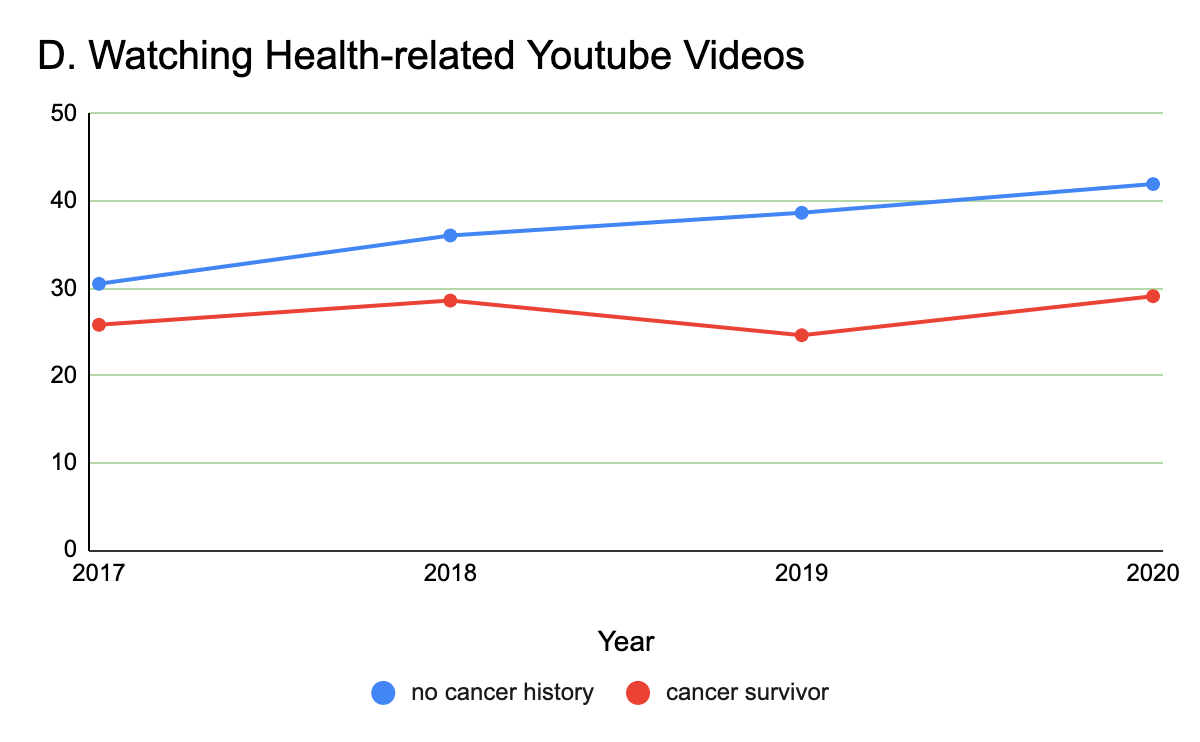


**Supplement Table 1: Sociodemographic Characteristics of Cancer Survivors by Social Media Usage, HINTS 2017-2020**

| **Sociodemographic characteristics** | **Total (n=1,840)**  **%**^†^ | **Social media non-users**  **(n= 1,175)**  **%**^†^ | **Social media**  **users**  **(n= 665)**  **%**^†^ | **Test statistic** | **p-value** |
| --- | --- | --- | --- | --- | --- |
| **Gender** |  |  |  |  |  |
| Female | 57.92 | 54.04 | 64.13 | 7.2481 | 0.0077 |
| Male | 42.08 | 45.96 | 35.87 |  |  |
| **Age** |  |  |  |  |  |
| Young (<40 years) | 10.22 | 7.21 | 15.05 | 17.2719 | <0.0001 |
| Middle aged (40-59 years) | 30.64 | 23.07 | 42.79 |  |  |
| Older (60+ years) | 59.13 | 69.72 | 42.15 |  |  |
| **Race** |  |  |  |  |  |
| Non-Hispanic White | 79.65 | 84.06 | 73.04 | 3.1058 | 0.0316 |
| Non-Hispanic Black | 8.19 | 6.80 | 10.26 |  |  |
| Hispanic | 8.06 | 6.22 | 10.83 |  |  |
| Other^‡^ | 4.10 | 2.93 | 5.86 |  |  |
| **Education** |  |  |  |  |  |
| High school or less | 27.65 | 33.59 | 18.20 | 8.0422 | 0.0007 |
| Some college | 39.81 | 35.90 | 46.03 |  |  |
| College graduate or postgraduate | 32.54 | 30.51 | 35.77 |  |  |
| **Income group** |  |  |  |  |  |
| Low (< $35,000) | 26.40 | 31.50 | 18.60 | 5.0401 | 0.01 |
| Middle ($35,000 to < $50,000) | 14.44 | 13.49 | 15.89 |  |  |
| High ($50,000+) | 59.16 | 55.01 | 65.51 |  |  |
| **Insurance status** |  |  |  |  |  |
| Uninsured | 3.56 | 3.54 | 3.60 | 0.0015 | 0.9688 |
| Insured | 96.44 | 96.46 | 96.40 |  |  |
| **Comorbidities** |  |  |  |  |  |
| None | 36.86 | 33.77 | 41.79 | 4.1223 | 0.0437 |
| At least one | 63.14 | 66.23 | 58.21 |  |  |
| **Residence** |  |  |  |  |  |
| Urban | 85.71 | 83.80 | 88.77 | 4.4678 | 0.0358 |
| Rural | 14.29 | 16.20 | 11.23 |  |  |
| **Access to a primary care provider** |  |  |  |  |  |
| No | 14.41 | 15.89 | 12.08 | 2.4271 | 0.1208 |
| Yes | 85.59 | 84.11 | 87.92 |  |  |

^†^ All numbers in the table are weighted column percentages

^‡^Other race includes non-Hispanic multiracial, non-Hispanic Asian, non-Hispanic American Indian or Alaskan native, Native Hawaiian or Pacific Islander
